# Can Short Tau Inversion Recovery (STIR) Imaging Be Used as a Stand-Alone Sequence To Assess a Perianal Fistulous Tract on MRI? A Retrospective Cohort Study Comparing STIR and T1-Post Contrast Imaging

**DOI:** 10.7759/cureus.52448

**Published:** 2024-01-17

**Authors:** Armeen Ahmad, Sudeep Roplekar, Anna Podlasek

**Affiliations:** 1 Radiology, Prime Hospital, Dubai, ARE; 2 Tayside Innovation MedTech Ecosystem (TIME), University of Dundee, Dundeee, GBR; 3 Radiological Sciences, University of Nottingham, Nottingham, GBR

**Keywords:** colorectal, imaging, mri, stir, perianal fistula

## Abstract

Introduction: Perianal fistulas demand precise preoperative assessment for optimal surgical outcomes. MRI, using Short Tau Inversion Recovery (STIR) and T1-post contrast sequences, plays a crucial role in this evaluation.This retrospective cohort study compared STIR imaging's diagnostic efficacy with T1-post contrast sequences in identifying perianal fistulous tracts. The study investigated whether STIR imaging could serve as the sole diagnostic sequence, simplifying clinical practice.

Methods: In a tertiary care hospital, 100 patients underwent pelvic MRI for suspected perianal fistulas. Radiologists independently evaluated STIR and T1-post contrast sequences for internal openings, tract extent, distinction, abscess presence, and tract type. Sensitivity, specificity, area under the curve (AUC), and Cohen's kappa analysis were used for diagnostic assessment.

Results: STIR imaging showed notable sensitivity (79.8-97.9%) and specificity (100%) for identifying internal openings and tracts. Combined with T1-post contrast, diagnostic accuracy improved significantly, with near-perfect AUC values. Kappa values indicated moderate to substantial agreement between radiological assessments and clinical diagnosis. The combined sequences achieved 100% sensitivity and specificity for tract visualization.

Conclusion: STIR imaging presents promise as a singular diagnostic tool for perianal fistulas, especially when combined with T1-post contrast sequences. While offering potential clinical diagnosis simplifications, further studies are warranted to validate its utility and ensure comprehensive diagnostic accuracy.

## Introduction

The anal sphincter, comprising the internal and external sphincters, differs notably in muscle composition and voluntary control. Damage to the external sphincter, a voluntary striated muscle, can lead to severe complications such as faecal incontinence. Therefore, accurately diagnosing and determining the anatomical extent preoperatively is critical for favourable surgical outcomes [1].

Perianal fistula, characterized by a tract connecting internally from the anal or rectal mucosa to the perianal skin, may manifest as low or high based on its internal opening location. Variations in its structure, including linear or curvilinear tracts with potential branching, inflammation, or abscess formation, underscore the complexity of this condition. Classifying systems such as Parks Classification (intersphincteric, transsphincteric, extrasphincteric, and suprasphincteric) and the St James's University Hospital classification further delineate the diverse presentations of perianal fistula [2,3].

Magnetic resonance imaging (MRI) is currently the standard imaging technique for accurately evaluating perianal fistulae [4]. For patients presenting with suspected perianal fistula, distinguishing between genuine fistulous tracts and non-fistulous conditions mimicking similar symptoms is crucial. MRI sequences like Short Tau Inversion Recovery (STIR), T2-weighted imaging (T2), T1-weighted imaging (T1), and post-contrast scans (C) in different planes are routinely used for evaluation, with several studies highlighting the significance of these sequences in comprehensive assessment [5-7].

Our study aimed to evaluate the efficacy of the STIR sequence compared to post-contrast T1WI in diagnosing perianal fistulas. Specifically, we sought to determine whether the STIR sequence could serve as a singular diagnostic tool for this condition, proposing its potential as a stand-alone sequence in clinical practice.

## Materials and methods

This single-arm, retrospective, observational cohort study included 100 participants. It was conducted from January 2020 to March 2023 in a tertiary care hospital, Prime Hospital, Dubai, in the United Arab Emirates (UAE). The Ethics Committee at Prime Hospital, Dubai, UAE, approved the study on June 7, 2023 (approval number: 07/06/2023).

All consecutive patients who were clinically suspected of perianal fistula (perianal discharge, pelvic pain with no visible external opening as per individual decision of the referring physician) and underwent MRI of the pelvis were included in this study. There was no restriction on gender or age; both outpatients and inpatients were included. The exclusion criteria were claustrophobia and/or inability to undergo MRI.

The study was performed on a 1.5 Tesla MRI (MAGNETOM Essenza; Siemens Healthineers AG, Forchheim, Germany). The initial sagittal image was taken, and axial and coronal cuts were planned on the sagittal image. Coronal sections were planned parallel to the anal canal.

The routine departmental protocol of pelvic MRI included the following sequences: (i) STIR in three planes, (ii) sagittal T2 thin section, (iii) coronal T1, (iv) T1W fat-sat pre-contrast, (v) post-contrast T1WI in three planes. Since our study focused on comparing perianal fistula detection between STIR and T1C sequences, we only focused on comparing these two. The detailed MRI acquisition protocol is summarized in Table 1.

**Table 1:** MRI acquisition protocol MRI acquisition protocol for T1-weighted imaging post-contrast (T1C) and STIR STIR: Short Tau Inversion Recovery; FOV: field of view; TR/TE=repetition time/time to echo

Sequence	Imaging Plane	FOV (cm)	TR/TE	Slice Thickness (mm)	Inter Slice gap (mm)	Matrix
T1C	Axial	260	540/9.1	3	10	192x256
T1C	Coronal	280	496/9.1	3	10	182x265
T1C	Sagittal	260	500/9.1	3	10	192x256
STIR	Axial	285	6670/34	3	10	205x256
STIR	Coronal	285	5384/34	3	10	205x256
STIR	Sagittal	235	3920/35	3	10	205x256

Two radiologists with more than 10 years of experience read the cases independently and compared only STIR and TIC images. An agreement was reached on the following assessed parameters: (i) internal opening, (ii) extent of the tract, (iii) tract distinction, (iv) presence or absence of an abscess, (v) type of tract. If more than one abscess was present, the characteristics of one were described in a single patient. 

The manuscript follows the Strengthening the Reporting of Observational Studies in Epidemiology (STROBE) guidelines [8].

Statistical analysis

The baseline statistics were presented as numbers and percentages or mean and standard deviation (SD), as appropriate. We investigated the diagnostic performance of MRI sequences using sensitivity, specificity, area under the curve (AUC), and receiver operating characteristic (ROC) analysis. Sensitivity refers to the test's ability to correctly identify individuals with the condition, while specificity measures its capacity to identify those without the condition accurately. AUC, derived from the ROC curve, provides a single scalar value representing the overall discriminatory ability of the test; an AUC closer to 1 signifies excellent discriminative power. ROC analysis graphically illustrates the trade-off between sensitivity and specificity [9].

We also employed Cohen's kappa analysis as a robust statistical measure to evaluate agreement when assessing binary data between assessments, with which we were able to quantify the level of agreement beyond chance between MRI and clinical diagnosis, which achieves the value between -1 and 1 (Kappa result can be interpreted as follows: values ≤ 0 indicating no agreement and 0.01-0.20 as none to slight, 0.21-0.40 as fair, 0.41- 0.60 as moderate, 0.61-0.80 as substantial, and 0.81-1.00 as almost perfect agreement) [8]. Excel (Microsoft Corporation, Redmond, Washington, United States), IBM SPSS Statistics for Windows, Version 26.0 (Released 2019; IBM Corp., Armonk, New York, United States) and Statistica v 13.1 (Released 2017; Dell Inc., Round Rock, Texas, United States) were used for data analysis.

## Results

A total of 100 patients were evaluated by MRI who came with a clinical suspicion of perianal fistula. All eligible participants were included in the study, and due to the retrospective nature, there was no loss for follow-up. Baseline demographic characteristics are presented in Table 2. 

**Table 2:** Baseline demographic characteristics

Demographic characteristics	Value
Total number of patients, n	100
Age (years), mean±SD; (range)	38±11; (13-74)
Females, n (%)	26 (26%)
Males, n (%)	74 (74%)

**Figure 1:**
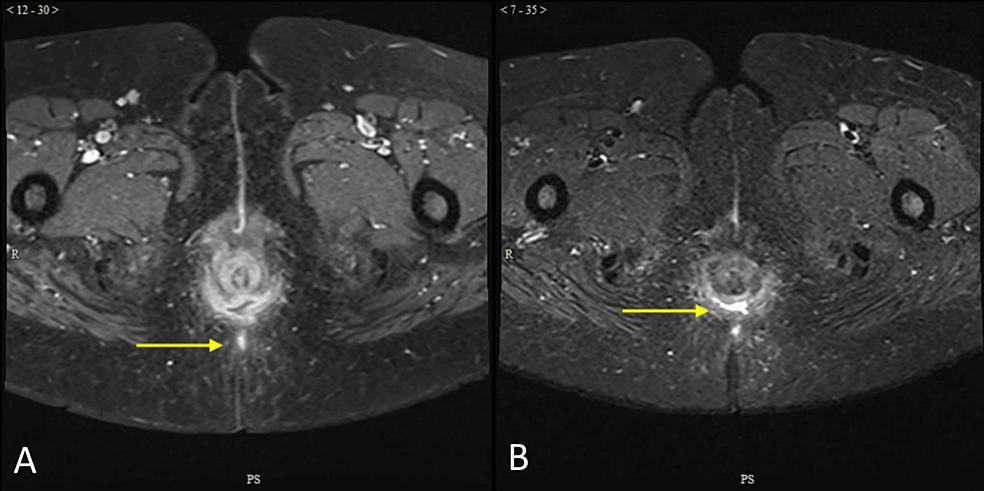
Horseshoe abcess on a T1C with the arrow indicating the tract (A) and STIR with the arrow indicating the abscess (B) T1C: post-contrast T1-weighted imaging; STIR: Short Tau Inversion Recovery

Out of the 100 suspected cases, six cases turned out to be normal on MRI. Of 94 tracts, 82 (87.2%) were low and 12 (12.8%) high tracts. Additionally, they were divided into seven (7.5%) extra-sphincteric, 66 (70.2%) inter-sphincteric, and 21 (22.3%) trans-sphincteric. The horseshoe was present in 11 (11.7%) cases. Regarding complication, the ramification of the tract was present in 15 (16%) cases, and the abscess was visible in 36 (38.3%) cases (Figure 1) 

**Figure 2:**
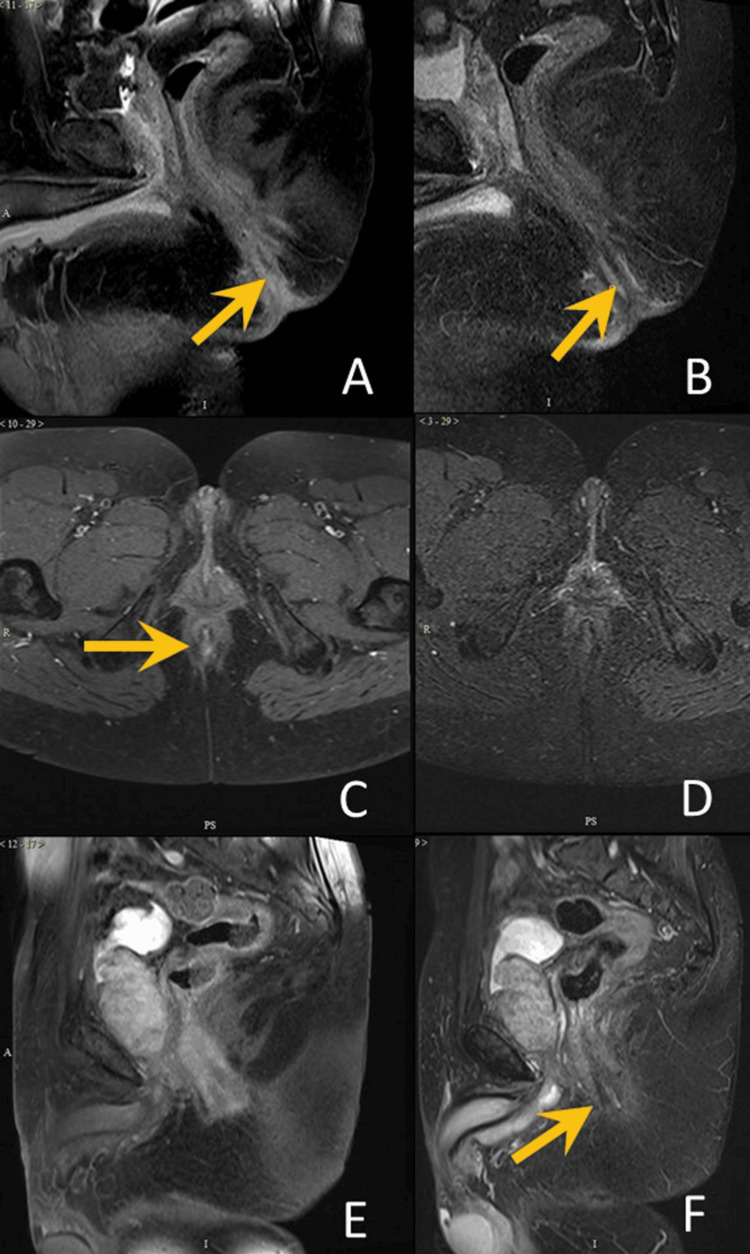
Visualization of abscess tract (i) Case 1: seen both on T1C (A) and STIR (B) (ii) Case 2: seen on T1C (C), but not on STIR (D) (iv) Case 3 – not seen on T1C (E), but seen on STIR (F) T1C: post-contrast T1-weighted imaging; STIR: Short Tau Inversion Recovery

The tract was always visualized on MRI. In 83 (88.3%) cases, it was visible in both sequences. Out of 11 cases, nine (81.8%) visualized only on STIR and 2 (18.2%) cases on T1C. Agreement between the radiology score and clinical diagnosis was kappa 0.466, p<0.001. (Figure 2)

**Figure 3:**
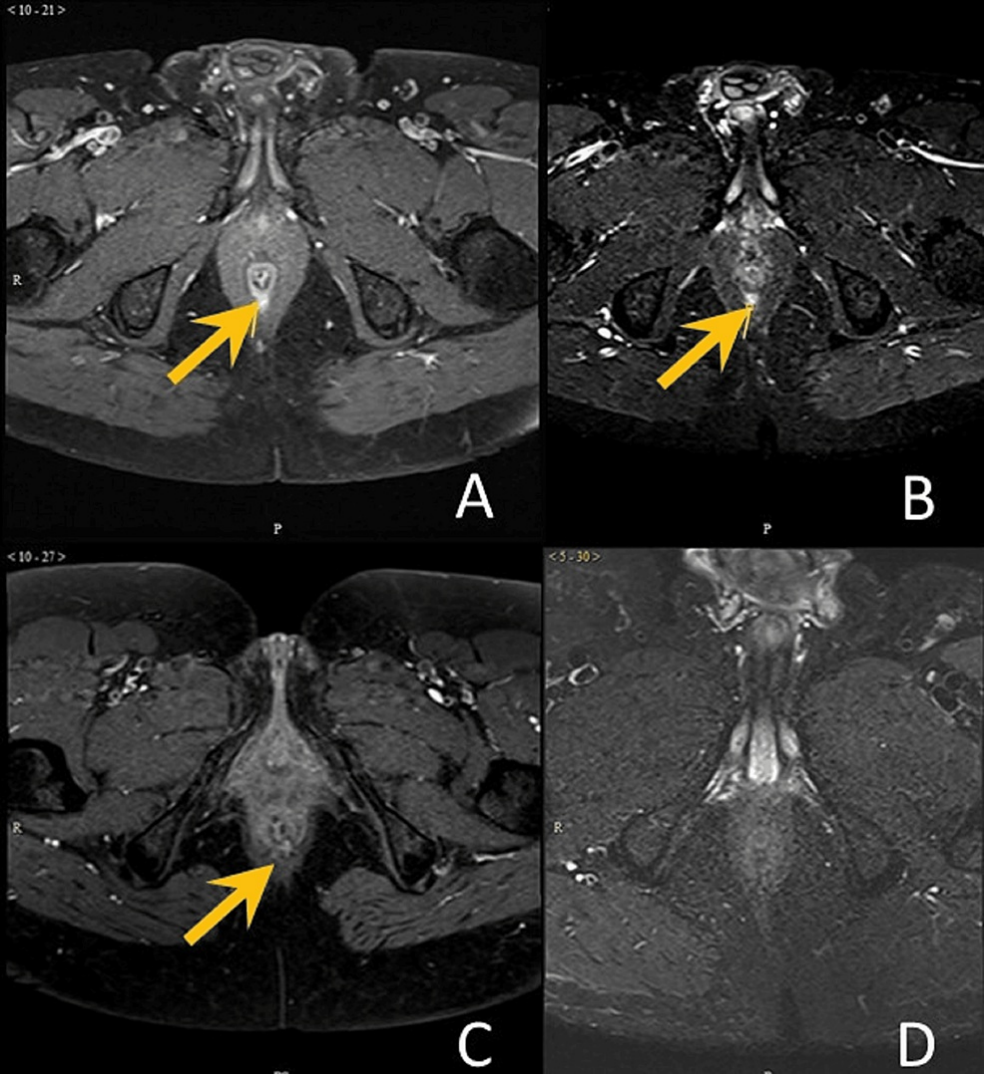
Visualization of internal opening (i) Case 1: seen both on T1C (A) and STIR (B) (ii) Case 2: seen on T1C (C), but not on STIR (D) T1C: post-contrast T1-weighted imaging; STIR: Short Tau Inversion Recovery

The internal opening was visible concomitantly on both sequences in 63 (67%) cases and was absent in seven (7.5%). Out of the remaining 24 cases, 12 (50%) were visualized only on STIR and 12 (50%) only on T1C. Agreement between the radiology score and clinical diagnosis was kappa 0.340, p<0.001 (Figure 3)

**Figure 4:**
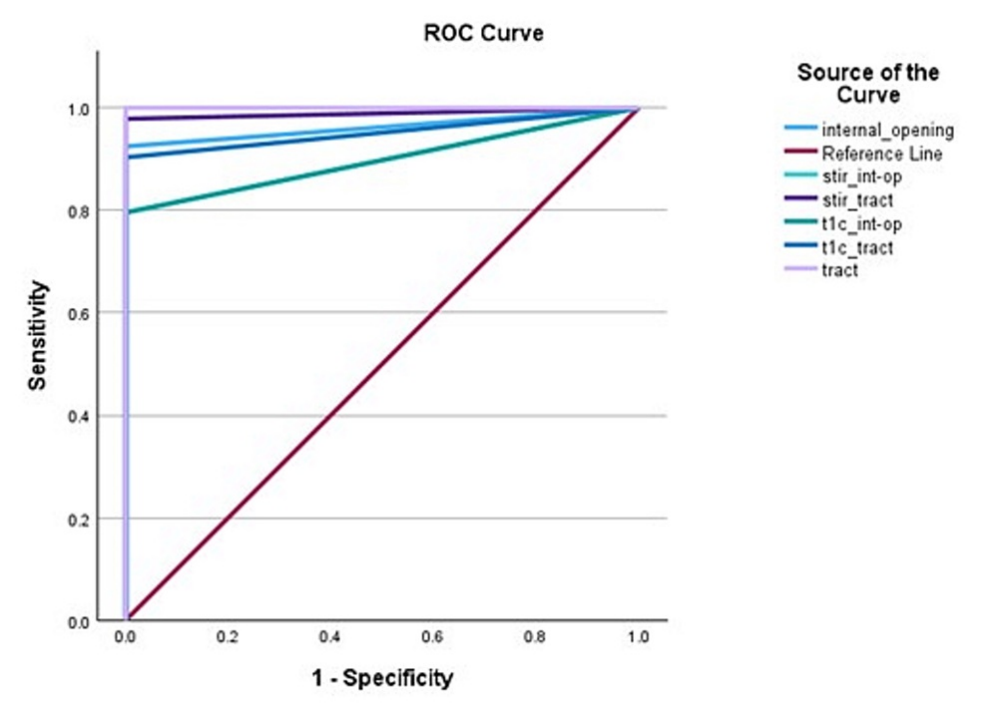
ROC curve of imaging features (internal opening, tract) on MRI sequences – individually or combined ROC: receiver operating characteristic; T1C: post-contrast T1-weighted imaging; STIR: Short Tau Inversion Recovery (STIR); int-op = internal opening

Table 3 and Figure 4 summarize the sensitivity, specificity, AUC, and kappa values for the imaging features on single or combined MRI sequences in opposition to the clinical diagnosis.

**Table 3:** Summary results The summary of sensitivities, specificities, AUC and Kappa values for the imaging features on single or joint MRI sequences versus clinical gold truth STIR: Short Tau Inversion Recovery; T1C=post-contrast T1-weighted imaging; AUC: area under the curve

	Sensitivity	Specificity	AUC	Kappa
Internal opening – STIR	79.8%	100%	0.899	0.285
Internal opening – T1C	79.8%	100%	0.899	0.321
Internal opening - combined	92.6%	100%	0.962	0.599
Tract – STIR	97.9%	100%	0.989	0.847
Tract – T1C	90.4%	100%	0.952	0.531
Tract - combined	100%	100%	1.000	1.000

## Discussion

In our study, a subset of patients who presented with perianal pain where the external opening was not visible clinically were referred for an MRI study to assess the possibility of an underlying fistula.

The main difference between T1 post-contrast and STIR sequences in MRI imaging for perianal fistulas is their ability to assess different aspects of the condition. T1 post-contrast sequences are used to assess abnormal enhancement related to inflammation. This sequence allows for the differentiation of abscess or fluid within a fistula track from granulation tissue, as an abscess demonstrates rim enhancement with central nonenhancement. Contrast injection adds cost and time but provides valuable information. On the other hand, STIR sequences are used to assess oedema and increase the conspicuity of inflammatory changes related to an active fistula on the scan. Inflammatory changes may extend beyond the fistula itself, particularly in the context of a contained abscess. This sequence allows for the visualization of the anatomy of the anal canal and the identification of internal and external sphincter muscles, as well as fibrosis at the site of a healed fistula [10].

The evaluation of the imaging sequences demonstrated substantial sensitivity (79.8-97.9%) and specificity (100%) for detecting features like internal openings and tracts. The individual assessments using STIR and T1C sequences revealed promising diagnostic performance, with combined sequences showcasing improved sensitivity and AUC values approaching 1, indicating excellent discriminative ability. Kappa values, indicative of agreement between radiological assessments and clinical diagnosis, ranged from moderate to substantial, reinforcing the efficacy of MRI in identifying internal openings and tracts. The ROC curve analysis further supported the diagnostic accuracy of the MRI sequences, highlighting their capability to discern internal openings and tracts. Notably, combining both sequences yielded optimal results, achieving 100% Sensitivity and Specificity for tract visualization.

In a previous study by Halligan et al., in the subset of patients with more severe disease and suspected of perianal sepsis, STIR sequences alone were sufficient for diagnosing and classifying fistula-in-ano [11]. The study also found that T1 weighted sequences were generally non-contributory in diagnosis. However, other studies looking into consecutive patients clinically suspected of perianal fistula showed a high sensitivity of 96.6% and a diagnostic accuracy of 90.6% of STIR sequence compared to surgical findings as the gold standard [4]. The specificity of STIR for perianal fistula was reported to be moderate at 67.7% in this study [4], which significantly differs from our results.

For the identification of a fistulous tract, STIR imaging was found to be highly sensitive [11], while the combination of STIR and T1 post-contrast imaging was found to be more sensitive in identifying the internal opening. Stand-alone STIR imaging could be offered as an excellent negative predictive value to exclude/rule out the possibility of a fistulous tract [12], especially in patients who were unable to lie in the MRI scanner for long due to pain/claustrophobia, who cannot be given IV contrast due to renal impairment, or who may have financial constraints.

Furthermore, whenever possible, combined use of STIR and post-contrast T1 weighted imaging can delineate the tract, internal opening and complications of perianal fistula [7]. This can help the surgeon in decision-making prior to surgical repair.

Despite its significant contributions, this study has several limitations that warrant consideration. Its retrospective design and reliance on patients who underwent MRI for suspected perianal fistulas may introduce selection bias and limit the representation of less typical cases or those managed without imaging. With a sample size of 100 from a single centre, the generalizability of findings might be restricted. Interobserver variability among radiologists and the potential exclusion of specific patient demographics due to claustrophobia or inability to undergo MRI could impact the study's comprehensiveness and applicability. Furthermore, the study lacks comparison with a gold standard, such as surgical findings, which might limit the ability to determine the diagnostic accuracy of the MRI sequences. Focusing solely on STIR and T1C sequences overlooks the potential benefits of other MRI sequences, potentially limiting a comprehensive evaluation. Thus, while promising, further prospective studies involving more extensive and diverse populations, multi-centre collaborations, and comparison with gold standards are warranted to validate these MRI sequences' practical utility and reproducibility in diagnosing perianal fistulas in clinical practice.

## Conclusions

The study assessed the effectiveness of STIR imaging versus T1-post contrast imaging in diagnosing perianal fistulas through MRI evaluation. It revealed both sequences' significant sensitivity and specificity in identifying crucial fistula features like internal openings and tracts. STIR imaging demonstrated high sensitivity (79.8-97.9%) and promising diagnostic accuracy, especially when combined with T1-post contrast sequences, showcasing excellent discriminative ability.

The research proposes STIR imaging as a potentially stand-alone diagnostic tool for perianal fistulas, particularly for visualizing the fistulous tract. This could prove beneficial for patients facing limitations like claustrophobia or financial constraints. Additionally, using STIR and T1-post contrast sequences enhances internal opening visualization, tract delineation, and complication assessment, aiding surgical planning.

## References

[1] Balcı S, Onur MR, Karaosmanoğlu AD, Karçaaltıncaba M, Akata D, Konan A, Özmen MN (2019). MRI evaluation of anal and perianal diseases. Diagn Interv Radiol.

[2] Morris J, Spencer JA, Ambrose NS (2000). MR imaging classification of perianal fistulas and its implications for patient management. Radiographics.

[3] Daabis N, El Shafey R, Zakaria Y, Elkhadrawy O (2013). Magnetic resonance imaging evaluation of perianal fistula. Egypt J Radiol Nucl Med.

[4] Jabeen N, Qureshi R, Sattar A, Baloch M (2019). Diagnostic accuracy of short tau inversion recovery as a limited protocol for diagnosing perianal fistula. Cureus.

[5] Varsamis N, Kosmidis C, Chatzimavroudis G (2023). Preoperative assessment of perianal fistulas with combined magnetic resonance and tridimensional endoanal ultrasound: a prospective study. Diagnostics (Basel).

[6] Gage KL, Deshmukh S, Macura KJ, Kamel IR, Zaheer A (2013). MRI of perianal fistulas: bridging the radiological-surgical divide. Abdom Imaging.

[7] Zhao WW, Yu J, Shu J (2023). Precise and comprehensive evaluation of perianal fistulas, classification and related complications using magnetic resonance imaging. Am J Transl Res.

[8] Cuschieri S (2019). The STROBE guidelines. Saudi J Anaesth.

[9] Fardy JM (2009). Evaluation of diagnostic tests. Methods Mol Biol.

[10] Tolan DJ (2016). Magnetic resonance imaging for perianal fistula. Semin Ultrasound CT MR.

[11] Halligan S, Healy JC, Bartram CI (1998). Magnetic resonance imaging of fistula-in-ano: STIR or SPIR?. Br J Radiol.

[12] Lo Re G, Tudisca C, Vernuccio F (2016). MR imaging of perianal fistulas in Crohn's disease: sensitivity and specificity of STIR sequences. Radiol Med.

